# ABC and IFC: Modules Detection Method for PPI Network

**DOI:** 10.1155/2014/968173

**Published:** 2014-06-02

**Authors:** Xiujuan Lei, Fang-Xiang Wu, Jianfang Tian, Jie Zhao

**Affiliations:** ^1^School of Computer Science, Shaanxi Normal University, Xi'an, Shaanxi 710062, China; ^2^School of Electronics Engineering and Computer Science, Peking University (Visiting Scholar), Beijing 100871, China; ^3^Division of Biomedical Engineering, University of Saskatchewan, Saskatoon, SK, Canada S7N 5A9

## Abstract

Many clustering algorithms are unable to solve the clustering problem of protein-protein interaction (PPI) networks effectively. A novel clustering model which combines the optimization mechanism of artificial bee colony (ABC) with the fuzzy membership matrix is proposed in this paper. The proposed ABC-IFC clustering model contains two parts: searching for the optimum cluster centers using ABC mechanism and forming clusters using intuitionistic fuzzy clustering (IFC) method. Firstly, the cluster centers are set randomly and the initial clustering results are obtained by using fuzzy membership matrix. Then the cluster centers are updated through different functions of bees in ABC algorithm; then the clustering result is obtained through IFC method based on the new optimized cluster center. To illustrate its performance, the ABC-IFC method is compared with the traditional fuzzy C-means clustering and IFC method. The experimental results on MIPS dataset show that the proposed ABC-IFC method not only gets improved in terms of several commonly used evaluation criteria such as *precision*, *recall*, and *P* value, but also obtains a better clustering result.

## 1. Introduction

With the completion of human genome project, researches on protein-protein interaction (PPI) networks [1] have been a hot topic in the life science area, which can not only provide clues to explore biological functions to deeply understand the essence of life activities, but also give important information to understand mechanism of diseases. Currently, a number of methods have been proposed in order to detect protein functional modules and predict protein functions from PPI networks, such as Markov random field method and spectral clustering method [2]. However, PPI networks are complex because of enormous data volume. Furthermore, PPI networks usually consist of a large number of high-density protein nodes and some sparse connection nodes, which causes the networks to show the small-world and scale-free features. Hence, clustering is the primary tool for data mining in PPI networks.

Clustering methods are typical tools in data mining. Recently many clustering methods emerged [3], such as traditional partitioning methods [4], which can only find the globular cluster and, meanwhile, the cluster number should be known in advance, hierarchical methods [5–8] which are computationally expensive and are difficult in determining the merging threshold, density-based methods [9–13] which cannot categorize the network that has a large number of sparse nodes, MCL-based methods [14] which are suitable to hard clustering, spectral clustering methods [15] in which it is difficult to determine the neighborhood matrix, fuzzy clustering methods [16] which are sensitive to initial value and are easy to fall into local minimum, and so on. Functional flow clustering algorithm [17, 18] is a new clustering method for PPI networks, which regards each node as a “reservoir” and passes on the flow to the next node by the connecting edge. The algorithm is easy to be implemented and suitable for PPI networks. But the* precision* and* recall* values are not good enough. Thus it can be seen that these methods have different degrees of drawbacks in mining PPI networks due to the unique characteristics of PPI networks. We have done some researches on PPI networks clustering issues based on swarm intelligent optimization methods [19–21], but the performance of the algorithms still needs to be improved.

Some classical clustering algorithms split samples into some disjoint clusters; that is, one sample belongs to only one cluster absolutely. Results of these methods are not ideal for PPI networks in that there are some overlapping functional modules in PPI networks. In fact, proteins in PPI networks do not belong to a single cluster. According to the definition of fuzzy partition, Dunn [22] has extended hard clustering to fuzzy clustering, which allows one protein to belong to multiple clusters. This expresses the fuzzy concept of “it belongs to both this cluster and that cluster.” Therefore, fuzzy clustering algorithm is more suitable for clustering proteins in PPI networks. Fuzzy c-means (FCM) clustering method [23], optimal fuzzy clustering algorithm [24], and intuitionistic fuzzy clustering (IFC) [25] algorithm are commonly used. Traditional fuzzy clustering algorithms adopt distance as the measure of sample similarity criteria. As most nodes in PPI networks are unreachable, it is difficult to apply them to PPI networks effectively.

In this paper a new clustering model is proposed by combining artificial bee colony (ABC) optimization and intuitionistic fuzzy membership. Specifically, the proposed methods employ fuzzy membership matrix for partitioning initial clusters first. Then, a new clustering objective function is presented by taking characteristics of PPI networks, the similarity among clusters, and the density and the weights of interaction nodes within clusters into consideration. The new method also takes advantage of ABC algorithm to optimize the values of the objective function to gain the cluster results. In addition, the new algorithm makes use of ABC algorithm to optimize cluster centers automatically to overcome defects of sensitivity to cluster centers through fuzzy c-means clustering and intuitionistic fuzzy clustering algorithm. Computational experiments show that the new model and algorithm perform better than existing algorithms.

The rest of this paper is organized as follows. In Section 2, basic concepts and principles are introduced firstly; secondly the proposed ABC fuzzy clustering model is discussed, and then the flow chart and the computational step are listed, along with the time complexity analysis of the algorithm. Performance and evaluation of the ABC-IFC algorithm is shown by comparing with FCM and IFC in Section 3.  Section 4 concludes this study.

## 2. Materials and Methods

### 2.1. Two Properties of PPI Networks

Watts and Strogatz [26] presented small-world property of complex networks, stating that most nodes are not neighbors of one another, while they can be reached from any of other nodes by a small number of hops or steps. A small-world network has smaller average distance than random networks and larger clustering coefficient than regular networks. There are some subnetworks in which almost any two nodes are connected because of the effect of the large clustering coefficient. On the other hand, any two nodes are connected mostly through one short path.

Regarding each network as an undirected graph, the *e* degree of node *v* is called *k* if node *v* has *k* adjacent node. *P*(*k*) represents the frequency of nodes with the degree of *k*; if *P*(*k*) follows the power-law distribution such as *P*(*k*) ∝ *k* ^−*γ*^, with *γ* ∈ [2,3], the graph is considered scale-free. With scale-free property [27], only the degree of a small number of nodes is large while that of most nodes is small. Statistical physicists used to call the phenomenon following power-law distribution as scale-free phenomenon.

### 2.2. Fuzzy C-Means Clustering

Fuzzy C-means clustering (FCM) algorithm is described by Ruspini [28]. It is defined as follows: *X* = {*x*_1_, *x*_2_,…, *x*_*n*_} is cluster sample, *n* is the number of samples, *c* is the number of clusters, 1 < *c* < *n*, *d*_*ij*_ = ||*x*_*i*_ − *v*_*j*_|| is Euclidean distance between the sample *x*_*i*_ and the cluster center *v*_*j*_, *V* = [*v*_1_, *v*_2_,…, *v*_*c*_] is cluster center matrix, *U* = [*u*_*ij*_] is *n*∗*c* membership matrix, and *u*_*ij*_ is the degree of membership of *x*_*i*_ that belongs to the cluster *j*. Update the degree of membership *u*_*ij*_ and the cluster center *v*_*j*_ by

(1) $$\begin{array}{c} u_{ij}=\frac{1}{\sum_{t=1}^{c}{\left({||x_{i}-v_{j}||}^{2}/{||x_{i}-v_{t}||}^{2}\right)}^{2/\left(m-1\right)}}\\ v_{j}=\frac{\sum_{i=1}^{n}u_{ij}^{m}x_{i}}{\sum_{i=1}^{n}u_{ij}^{m}}, \end{array}$$

where *m* = 2. The objective function is defined as

(2) $$\begin{array}{c} J=\overset{n}{\underset{i=1}{\sum }}\overset{c}{\underset{j=1}{\sum }}u_{ij}^{m}{||x_{i}-v_{j}||}^{2}; \end{array}$$

*J* will obtain the extremum when the matrix *U* is normalized.

### 2.3. Intuitionistic Fuzzy Clustering (IFC)

#### 2.3.1. Intuitionistic Fuzzy Set Theory

Let *X* be a given domain; an intuitionistic fuzzy set *R* on *X* is given by

(3) $$\begin{array}{c} R=\left\{\langle x,\mu_{R}\left(x\right),\nu_{R}\left(x\right)\rangle \;|\;x\in X\right\}, \end{array}$$

where *μ*_*R*_(*x*) : *X* → [0, 1] and *ν*_*R*_(*x*) : *X* → [0,1] denote degree of membership and nonmembership of *R*, respectively. For *x* ∈ *X* in *R*, it is true that 0 ≤ *μ*_*R*_(*x*) + *ν*_*R*_(*x*) ≤ 1. The general intuitionistic fuzzy set is denoted by *R* = 〈*x*, *μ*_*R*_(*x*), *ν*_*R*_(*x*)〉. The general intuitionistic fuzzy set is also marked as *R* = {〈*x*, *μ*_*R*_(*x*), 1 − *μ*_*R*_(*x*)〉 | *x* ∈ *X*}. *π*_*R*_(*x*) = 1 − *u*_*R*_(*x*) − *ν*_*R*_(*x*) denotes intuitionistic index of *x* in *R*, which is a measurement of uncertain degree.

#### 2.3.2. Intuitionistic Fuzzy Clustering Algorithm

Let *X* = {*x*_1_, *x*_2_,…, *x*_*n*_} be a set of samples to be clustered; *P* = {*p*_1_, *p*_2_,…, *p*_*c*_} is a set of *c* clustering prototypes, where *c* is the number of clusters. The intuitionistic fuzzy set is given by *R* = {〈(*x*_*i*_, *p*_*j*_), *μ*, *ν*〉 | *x*_*i*_ ∈ *X*, *p*_*j*_ ∈ *P*}; (*x*_*i*_, *p*_*j*_) is the degree of membership of the sample *i* in the cluster *j*. *V* = [*v*_1_, *v*_2_,…, *v*_*c*_] is a group of cluster centers. Clustering prototype model (*p*_*ij*_), degree of hesitation (*π*_*ij*_), degree of membership (*u*_*ij*_), and degree of nonmembership (*v*_*ij*_) can be computed as follows:

(4) $$\begin{array}{c} p_{ij}=\alpha \left(1-e^{\left(d{\left(x_{i},p_{j}\right)}^{2}\right)/4}\right) \end{array}$$

(5) $$\begin{array}{c} e_{\lambda k}=\frac{1}{\sum_{i=1}^{c}\left(1-\pi_{ki}/e^{{d^{\left(x_{k},v_{i}\right)}}^{2}}+e_{\lambda k}\right)} \end{array}$$

(6) $$\begin{array}{c} \pi_{ij}=\{\begin{array}{cc} w_{e}\left(i,v_{j}\right) & \text{if}\;\;i\neq v_{j}\\ 1 & \text{otherwise} \end{array} \end{array}$$

(7) $$\begin{array}{c} u_{ij}=\frac{\left(1-\pi_{ij}\right)}{\left(e^{d{\left(x_{i},p_{j}\right)}^{2}}+1\right)} \end{array}$$

(8) $$\begin{array}{c} v_{ij}=1-\pi_{ij}-u_{ij}, \end{array}$$

where *α* = 0.4. *e*_*λk*_ in (5) is solved according to the fast secant method. In intuitionistic fuzzy clustering algorithm, the degree of hesitation is always set in advance. In this paper, the degree of hesitation *π* is determined according to the PPI network nodes interaction weight *w*_*e*_.

Clustering analysis can be converted to optimization problem which aims at minimizing the objective function (clustering criterion function) *J*:

(9) $$\begin{array}{c} J=\overset{n}{\underset{i=1}{\sum }}\overset{c}{\underset{j=1}{\sum }}u_{ij}d\left(x_{i},p_{j}\right); \end{array}$$

*d*(*x*_*i*_, *p*_*j*_) = (*x*_*i*_ − *p*_*j*_)(*x*_*i*_ − *p*_*j*_)^*T*^ is the square of Euclidean distance between the sample *i* and clustering prototype*j* and must satisfy equation *u*_*ij*_ + *v*_*ij*_ + *π*_*ij*_ = 1.

### 2.4. The ABC Algorithm

Artificial bee colony algorithm is an intelligent optimization algorithm proposed by Karaboga et al. [29–31] to solve the multivariate function optimization problems based on the intelligent behavior of honey bee swarm. Srinivasa Rao et al. [32] used it to solve network reconfiguration problem in distributed systems. Other researchers proposed chaotic artificial bee colony [33] and discrete artificial bee colony [34] algorithms. The artificial bee colony has become a research hotspot due to its simple idea, fast convergence rate, and superior performance. Our team had adopted it to optimize the threshold of clustering problem and achieved good results [35].

The ABC algorithm is a new metaheuristic bionic algorithm, where each bee can be viewed as an agent, and swarm intelligence is achieved through the cooperation between the individuals. The ABC algorithm includes two different mechanisms consisting of foraging behavior and propagating behavior. The ABC algorithm based on propagating mechanism was inspired by marriage behavior of bee colony [36]; the queen maintains good genes to make colony more adaptive to the environment. While the ABC algorithm based on foraging mechanism finds optimal solution through collaboration between the various types of bees and role conversion mechanism, the ABC algorithm provides a new idea for the heuristic algorithm research and becomes one of the important research directions of solving complex optimization problems.

The ABC algorithm based on foraging behavior typically contains four groups: nectar source, employed bees, onlooker bees, and scouts bees. A nectar source corresponds to an employed bee; the position of a nectar source represents a possible solution of optimization problem and its value depends on many factors, such as the degree of proximity between the nectar source and honeycomb or the degree of nectar source concentration and so on. The fitness is usually used to describe the nectar source features. The employed bees associate with certain nectar source, carrying a lot of nectar source information that is related to income level. The onlooker bees search and update new nectar source near honeycomb; if there are no nectar source for update, the scouts bees will search new nectar source in the global scope. Nectar source search follows two steps: firstly, the employed bees find nectar source and record the nectar source location and nectar quantity. Then the onlooker bees use the information the employed bees provided to decide which nectar source to go to, or scouts bees go on global searching to explore the new nectar source.

Based on foraging behavior, the ABC algorithm finds the global optimal value by each individual bees' local search, which has a faster convergence speed, higher* precision,* and few parameters. Therefore the ABC algorithm is used in this paper to optimize the IFC algorithm. The nectar sources stand for a set of clustering center of IFC algorithm; the cluster centers are optimized and updated by employed bees, onlookers bees, and scouts bees. It overcomes the sensitivity to clustering center of the IFC algorithm, while improving the effect of clustering.

### 2.5. ABC Fuzzy Clustering Model

#### 2.5.1. The Solution Space

The small-world and scale-free properties of PPI networks make few nodes have a large degree; these nodes may have an important impact on protein functions. Most of the other nodes own a relatively small degree, and even the degree of some nodes is zero which are named isolated nodes. In PPI networks, a protein may possess diverse functions; as a result, it is inadvisable to hold the idea that all the protein nodes in a cluster are regarded as having an identical function. Therefore, a lot of clustering methods without considering the characteristics perform unsatisfactory toward PPI networks. IFC clustering algorithm fits well with PPI networks of a protein belonging to several functional modules. As mentioned above, ABC algorithm performs admirably; we establish a corresponding relationship between clustering and the optimization mechanism of bee colony (Table 1). Accordingly, a model based on the combination of ABC optimization mechanism and intuitionistic fuzzy method is put forward in this paper. We name the model ABC intuitionistic fuzzy clustering mode (ABC-IFC, for short). The method takes advantage of ABC algorithm to determine the optimal cluster centers and overcomes the weakness of sensitivity to cluster centers by fuzzy c-means clustering and intuitionistic fuzzy clustering algorithm. Utilizing intuitionistic fuzzy algorithm to cluster PPI networks is expected to improve the clustering effect because the idea is in line with the characteristics of PPI networks.

#### 2.5.2. Principle of ABC Fuzzy Clustering Model

In the IFC clustering algorithm, a group of cluster centers are given randomly at the very beginning; afterwards the membership degree matrix is calculated and partitions are made according to it. Meanwhile the value of criteria is calculated until the algorithm satisfies the stopping condition and obtains the final clustering results. A set of initial cluster centers is generated randomly in IFC clustering algorithm, in which there are no rules to follow. Moreover the clustering criteria function is based on distance, which is unreasonable to deal with PPI networks. Hence, it is inefficient to cluster PPI networks by IFC clustering algorithm.

Since some nodes in PPI networks are unreachable, distance is not the suitable measure for clustering. As we know, there are interactions among nodes in PPI networks. We can compute the similarities among protein modules, density within modules, and average interactions using the interactions among the nodes. Accordingly, we redesign the criteria of IFC and evaluate the results in light of intermodule similarity, innermodule density, and similarity.

The new model ABC-IFC is proposed to seek the optimal cluster centers in this paper by combining IFC clustering algorithm and ABC algorithm. The ABC algorithm consists of three kinds of bees: employed bees, onlooker bees, and scout bees. In the ABC-IFC model, a nectar source stands for a group of cluster centers; the number of nectar sources is the number of clusters. Onlooker bees are responsible for exploiting new sources adjacent to employed bees and then updating the cluster centers. If onlooker bees fail to find the nectar source, scout bees will update cluster centers and search the whole area to revise the cluster centers. Finally, ABC algorithm will obtain the optimal cluster centers. IFC clustering algorithm will compute the fuzzy membership degree matrix based on the optimal cluster centers and, meanwhile, clusters are divided according to the membership degree matrix to obtain the clustering results in the end.

#### 2.5.3. Objective Function

Traditional clustering algorithms usually adopt distance to define the objective function; moreover in weighted networks, the reciprocal of the weights is regarded as the measure of distance between two nodes. The shortest path distance between two nodes is usually regarded as the distance when the two nodes are not connected. However, some nodes in PPI networks are unreachable, and the reciprocal of weights becomes infinite when there is no interaction between two protein nodes, so it is unreasonable to use the shortest path distance as the distance between two nodes. Thus, a new objective function is designed to evaluate the cluster results. Our objective function includes two parts: the first part is the similarity between interclusters; the second part is the reciprocal of the average of summation of inner cluster's density and the weights between nodes within a cluster. The less the first part and the second part, the better the performance will be. Thus the objective function is defined as

(10) min⁡ fval ={1c∗c∑i=1c∑j=1csim(Ii,Jj)+1DEWif    DEW≠01c∗c∑i=1c∑j=1csim(Ii,Jj)otherwise,

where sim(*I*_*i*_, *J*_*j*_) = (∑_*x*∈*I*_*i*_,*y*∈*J*_*j*__ *c*(*x*, *y*))/max⁡(|*I*_*i*_|, |*J*_*j*_|) stands for the similarity between two clusters,

(11) $$\begin{array}{c} c\left(x,y\right)=\{\begin{array}{cc} 1 & \text{if}\;\;x=y\\ w_{e}\left(x,y\right) & \text{if}\;\;x\neq y,\langle x,y\rangle \in E\\ 0 & \text{otherwise}, \end{array}\\ \text{den}\left(i\right)=\frac{2e}{n_{i}*\left(n_{i}-1\right)},\\ \text{dw}\left(i\right)=\frac{\text{sum}\left(w_{e}\right)}{n_{i}*\left(n_{i}-1\right)},\\ \text{DEW}=\frac{1}{c}\overset{c}{\underset{i=1}{\sum }}\left(\text{den}\left(i\right)+\text{dw}\left(i\right)\right); \end{array}$$

*n*_*i*_ represents the number of protein nodes in cluster *i*, *e* represents the number of interactions among the cluster and others in PPI networks, and  *w*_*e*_  represents the weights between nodes within a cluster.

The first part of (10) is similarity between two clusters, which is used to evaluate the similarity of two clusters; the smaller the similarity, the better the cluster effects will be, and vice versa. The second part is the reciprocal of the average of summation of inner cluster's density and the weights between nodes within a cluster, which reflects the strength of the interaction within a cluster. If there is no interaction between two nodes, the value of denominator of the second part may equal zero; that is, the second part tends to be infinitely small, and thus the first part becomes the criteria for cluster results. Otherwise, when the value of the second part is not zero, then the combination of similarity between two clusters, the density and weights within a cluster, can evaluate the cluster effect more comprehensively.

### 2.6. ABC-Based Fuzzy Clustering Algorithm

#### 2.6.1. Algorithm Description

**Step 1**

Initialize the iterations *iter* = 1, maximum iteration* maxiter*, and clustering number *c* and randomly select clustering center* V*, *limit* = 0, and *max*_*limit*. Set *gbestV* = *inf* initially*; gbestV *represents a group of optimal cluster centers;* gbest_cluster* is the clustering result of the optimal cluster centers which is set randomly at the beginning.

**Step 2**

Calculate the degree of membership matrix *U* using (5)–(7),* cluster* is formed according to the membership degree matrix, and evaluate the fitness value *fval*(*cluster*) based on (10).

**Step 3**

If *fval*  (*cluster*) < *fval*  (*gbest*_*cluster*), *gbestV* = *V* and *gbest*_*cluster* = *cluster*.

**Step 4**

Onlookers bees search new nectar source near employed bees; if the search is successful, go to Step 6.

**Step 5**

If the search is a failure and *limit* > *max* *limit*, scouts bees start global searching; set *limit* = 0.

**Step 6**

Update the clustering center *V*.

**Step 7**

Set *iter* = *iter* + 1.

**Step 8**

If *iter* < = *max* *iter*, go to Step 2, otherwise out put the cluster result *gbest*_*cluster*.

#### 2.6.2. Time Complexity of Algorithm

In time complexity analysis, asymptotic method is usually used to express the order of magnitude of program execution steps to estimate the performance of the algorithm. In ABC algorithm, if *c* represents cluster numbers, *n* is protein node, maximum iterations is* maxiter*, the population size is *N* which is twice the value of *c*, the node number of a class is *num*, and the time complexity of algorithm is as follows:

1. the time complexity of initializing the clustering center is *O*(*n*);
2. the time complexity of calculating the degree of membership matrix is *O*(*n*∗*c*);
3. the time complexity of updating the clustering center is *O*(*max* *iter*∗*N*∗*num*);
4. the time complexity of updating global optimal value is *O*(*N*);
5. the time complexity of cluster division is *O*(*n*∗*c*);
6. the time complexity of judging stopping condition is *O*(*max* *iter*).

From the above analysis we could find that the time complexity of (2) is dominating, the algorithm spends much time on the clustering center updating, which increases the time complexity.

## 3. Results and Discussions

### 3.1. Dataset and Criteria

In the experiments, the dataset of PPI networks comes from MIPS database [37], which consists of two sets of data: one is the experimental data which contains 1376 protein nodes and the 6880 interactive protein-pairs, which is considered the training database; the other describes the result that the proteins belong to identical functional module, which is regarded as the standard dataset [38], containing 89 clusters.

*Precision*,* recall*, *P* value, and their harmonic mean come to be the metric for evaluating the clustering in this paper.* Precision* [39] is the ratio of the maximal number of common nodes between experimental results and standard dataset to the number of nodes in experimental results.* Recall* [39] is the ratio of the maximal number of common nodes between experimental results and standard dataset to the number of nodes in the standard dataset. The equations are as follows:

(12) $$\begin{array}{c} precision\left(C\;|\;F\right)=\frac{\text{MMS}\left(C,F\right)}{\left|C\right|}\\ recall\left(C\;|\;F\right)=\frac{\text{MMS}\left(C,F\right)}{\left|F\right|}, \end{array}$$

where *C* stands for the obtained cluster results and *F* represents the standard dataset of MIPS. |*C*| is the number of nodes in the set *C*, |*F*| stands for the number of nodes in the standard dataset. MMS(*C*, *F*) denotes the number of the maximum matching nodes between experimental result and standard dataset.

A protein may possess various kinds of functions in PPI networks, so it is not advisable to consider that all the protein nodes in a cluster are regarded as having identical function. Thus, the definition of *P* value [40] comes to evaluate the reasonableness of this assignment. Suppose that the number of a cluster in the experiment is *m*, the number of protein nodes which possess identical function is *d*, the number of proteins in the standard database is*M*, and *D*is the number of proteins which have the same function as each other. The *P* value is expressed as follows:

(13) $$\begin{array}{c} P=\overset{m}{\underset{j=d}{\sum }}\frac{\left(\begin{array}{c} D\\ j \end{array}\right)\left(\begin{array}{c} M-D\\ m-j \end{array}\right)}{\left(\begin{array}{c} M\\ m \end{array}\right)}. \end{array}$$

According to (13), the lower the *P* value is, the more confident the protein nodes comes from one cluster module and possess identical function, which provides instructive information for researchers to analyze the function of unknown proteins.

In general, large modules own high* recall* value because a large module *C* contains a lot of nodes in the set of *F*, and extremely, all nodes are gathered in the same cluster; in this case the* recall* value tends to the highest. On the contrary, small modules possess high* precision* because small modules have the same properties. Extremely each node can be a module and these modules have the highest* precision*. Hence,* f-measure *comes to be the metric for clustering in this paper which is shown in (14). Consider

(14) $$\begin{array}{c} f\text{-}measure=\frac{2\cdot precision\cdot recall}{precision+recall}. \end{array}$$

### 3.2. Algorithm Parameter Analysis

In ABC algorithm, there are two important parameters, one is the* prob*, which is the probability that the onlooker bees select the nectar source, the other is the* limit*, which is the threshold indicating whether the scout bees will go on global searching.

Onlooker bees search the nectar source according to* prob*. A random number* rand*, arranged from 0 to 1, is generated firstly; if *rand* > *prob*, the onlooker bee chooses the node with the largest amount of information among the adjacent nodes of the employed bee to be the new cluster centers; otherwise, the node which has the second largest information is chosen to be the new cluster centers. Also* limit* is another important parameter in ABC algorithm. While there is no improvement of the cluster centers after* limit* times of loops, the cluster centers will be abandoned and the scout bees will search for a new solution of cluster centers.

**Figure 1:**
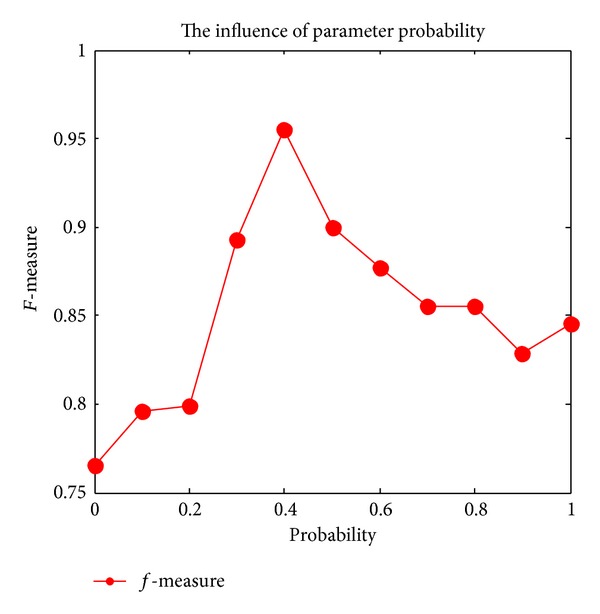
The influence of parameter *prob*.

Figure 1 shows the influence of parameter* prob* on the clustering results,* prob* is the parameter that onlooker bees select the nectar source according to the roulette wheel selection strategy. If the parameter is set too small, the possibility of onlooker bees searching local optimal clustering center is big and, meanwhile, the algorithm is easy to fall into local optimal. On the other hand, if it is too large, it could ensure the algorithm's diversity but onlooker bees could just find local suboptimal solution. From Figure 1, we find that the cluster effect is best when *prob* = 0.4.

**Figure 2:**
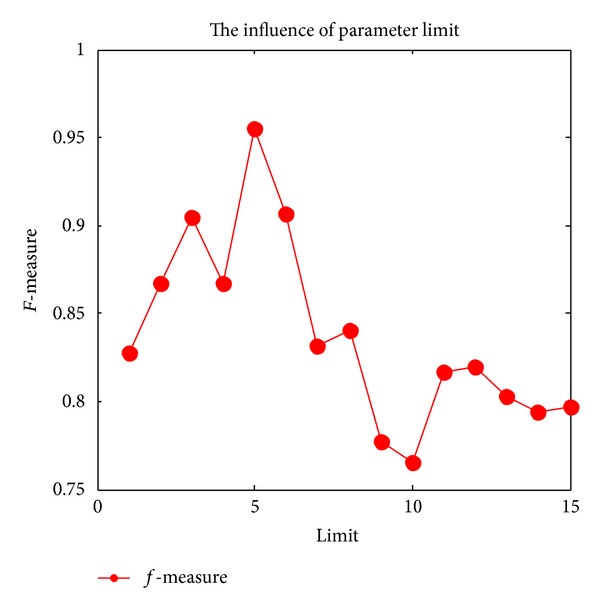
The influence of parameter* limit*.

Figure 2 shows the influence of parameter* limit* on the clustering results;* limit* is an important parameter in ABC algorithm which is used to determine whether the scout bees search the global area and update clustering center. From Figure 2, we can see that if* limit* is too small, the scout bees will constantly search new cluster centers to replace the current cluster centers, the algorithm will discard the optimal cluster centers. If* limit* is too large, the frequency of the scout bees searching the new solution will decrease and cause the algorithm to fall into local optimal and this will seriously affect the cluster results. Figure 2 show that when *limit* = 5, the algorithm reaches the best cluster results, and then when* limit* gradually increases, the cluster effect will gradually become poor.

### 3.3. Comparison of Performance among FCM, IFC, and ABC-IFC Algorithms

**Figure 3:**
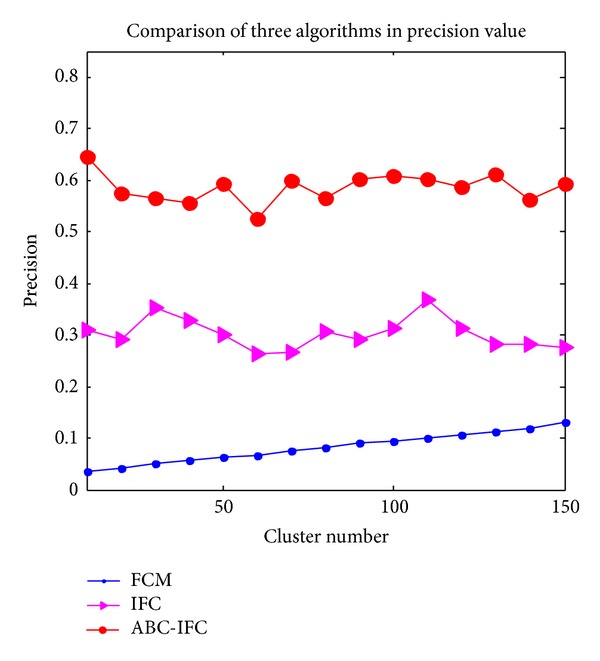
Comparison of three algorithms in* precision* value.

**Figure 4:**
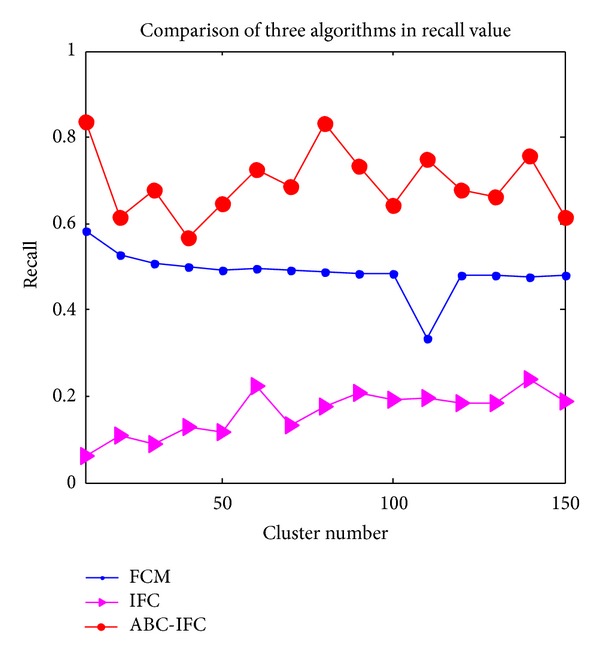
Comparison of three algorithms in* recall* value.

**Figure 5:**
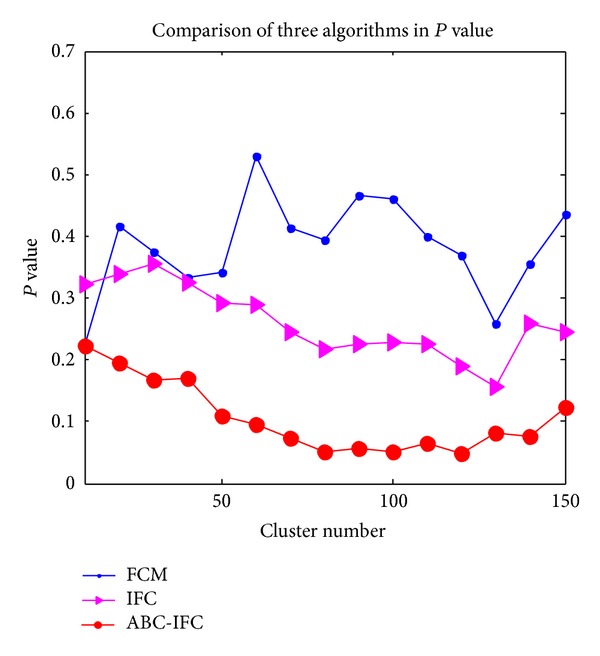
Comparison of three algorithms in *P*value.

**Figure 6:**
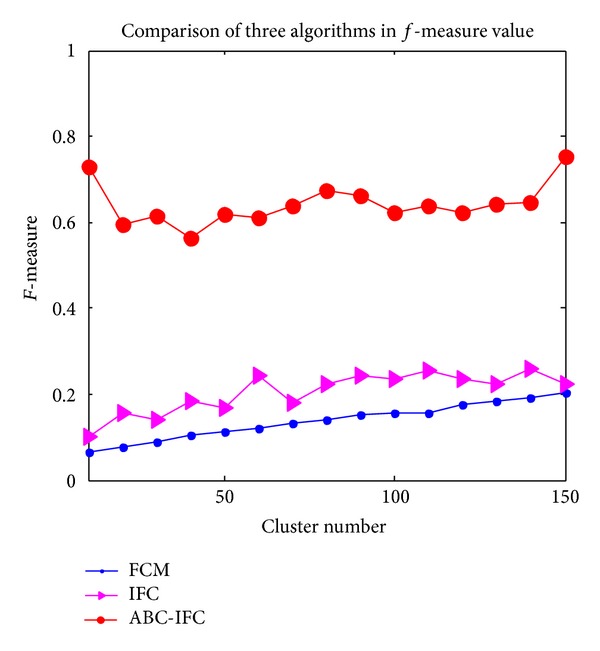
Comparison of three algorithms in* f-measure* value.

FCM clustering algorithm is very sensitive to the cluster centers; the performance of cluster results is poor. IFC algorithm is proposed based on fuzzy clustering, which performs better than FCM clustering, but the cluster effect is still not well enough. ABC-IFC algorithm overcomes the above drawbacks. Comparisons of* precision*,* recall*, *P* value, and* f-measure* of FCM algorithm, ICM algorithm and ABC-IFC algorithm are shown in Figures 3 to 6, respectively.

Figure 3 shows the comparison of* precision* of three algorithms. In Figure 3, it can be seen that ABC-IFC obtains the highest* precision *among three algorithms. The values of* precision* fluctuate largely when the number of clusters arranges from 20 to 70 and become steady and reach the best when the number of clusters is between 80 and 120. The* precision* values of IFC algorithm are between FCM and ABC-IFC algorithms; the highest* precision* is achieved when the cluster number is 110. Although* precision* tends to increase with the augment of cluster numbers in FCM algorithm, the* precision of FCM* is the lowest among three algorithms.

Figure 4 represents the comparison of* recall* value in three algorithms. Even if the values of ABC-IFC algorithm fluctuate strongly, they are the highest compared with the other algorithms. The values of FCM algorithm are higher than IFC but lower than ABC-IFC. From Figure 4, when the number of clusters is 110, the value tends to be the lowest.* Recall* of IFC is the worst among three algorithms.

Figure 5 describes the comparison of *P* value among three algorithms. *P* value of ABC-IFC decreases with the cluster number increasing at the beginning. *P* value increases gradually after the cluster number increases to 80. What is more, *P* value becomes lower and holds steady when the number of clusters arranges from 80 to 120. *P* value of IFC is between ABC-IFC and FCM. The highest value and strongest fluctuation of *P* value appears in FCM.

Comparison of* f-measure *among three algorithms is shown in Figure 6.* f-measure* of ABC-IFC is the highest among three algorithms and fluctuates slightly. Cluster results reach the best when the number of clusters changes from 80 to 90.* f-measure* of IFC is lower than ABC-IFC, in which* f-measure* tends to increase when the number of clusters increases, but the improvement is not obvious.* f-measure* of FCM increases with the clusters number increasing, but the values are far behind ABC-IFC. The cluster performance of IFC is better than FCM. However, ABC-IFC demonstrates excellent performance in terms of* precision*,* recall*, *P* value, and* f-measure*.

### 3.4. Cluster Results Analysis

Because it has a great impact on the algorithms, the number of cluster must be initialized first in ABC-IFC, IFC, and FCM algorithms. There is no rule to follow on determination of the number of clusters. Thus, the number of clusters tested was arranged from 10, 20, and 30 to 140 and 150.  Table 2 shows the comparison of clustering results with different cluster numbers. In FCM algorithm, running time is very short. When the cluster number increases,* recall* decreases, the* precision* and* f-measure* increase. Although the running time is short, the values of both* precision* and* f-measure* are too small so that FCM algorithm is not feasible. IFC algorithm performs better than FCM in terms of* precision*; the* recall* value is lower than in FCM and the running time is not quite long. But* precision*,* recall*, *P* value, and* f-measure* are not well enough. The running time of ABC-IFC algorithm amplifies with the increase of cluster numbers.* Precision*,* recall*, and *P* value are better than the FCM and IFC algorithms;* f-measure* reaches the highest and ABC-IFC performs steady with various cluster numbers.

Due to space limitations, Table 3 only listed the correctly and wrongly classified proteins in 8 clusters. It can be seen from Table 3 that the four clusters marked 1, 3, 6, and 8 are completely correct; clusters marked 2, 4, 5, and 7 are partly correct. Between the correctly and wrongly classified proteins, we can see that the proteins having the different functions provide a foundation for the research on the connection between different protein functional modules. The correctly classified proteins have the same function, which provides analysis basis for us to identify protein functional modules and predict protein function accurately.

## 4. Conclusions

In this paper, the fuzzy clustering algorithm has been used in PPI network clustering. Because there are some nodes unreachable in PPI network, we have redesigned the clustering objective function and proposed a novel clustering model combined with the optimization mechanism of artificial bee colony with the intuitionistic fuzzy clustering. The computational results on PPI dataset have shown that the algorithm could not only overcome the drawbacks of sensitivity to clustering center, but also have the highest accuracy and* recall* rate, the lowest *P* value, and the best* f-measure* among the competing algorithms. Meanwhile, the algorithm has a good effect on PPI network functional modules and also has great potential to solve other small-world and scale-free characterized complex network problems.

## Figures and Tables

**Table 1:** Corresponding relationship between clustering and mechanism of bee colony optimization.

Foraging behavior of honey bees	Clustering
Position of nectar sources	Cluster centers
Amount of nectar sources	Value of objective function
Responsibilities of onlooker bees and scout bees	Searching for optimizing cluster centers
Highest nectar amount of nectar sources	Best cluster centers

**Table 2:** The comparison of three algorithms on different cluster numbers.

Algorithm	Cluster number	Precision	Recall	*P*value	*F-*measure
FCM	10	0.0339	0.5814	0.2256	0.0641
IFC	10	0.3095	0.0606	0.3231	0.1014
ABC-IFC	10	0.6444	0.8373	0.2226	0.7283

FCM	20	0.0409	0.5269	0.4166	0.0759
IFC	20	0.2924	0.1074	0.3383	0.1571
ABC-IFC	20	0.5741	0.6156	0.1964	0.5941

FCM	30	0.0499	0.5080	0.3753	0.0909
IFC	30	0.3525	0.0883	0.3555	0.1412
ABC-IFC	30	0.5647	0.6789	0.1667	0.6166

FCM	40	0.0581	0.5006	0.3322	0.1041
IFC	40	0.3288	0.1288	0.3253	0.1851
ABC-IFC	40	0.5559	0.5691	0.1698	0.5624

FCM	50	0.0624	0.4905	0.3405	0.1107
IFC	50	0.3010	0.1172	0.2907	0.1687
ABC-IFC	50	0.5933	0.6452	0.1094	0.6182

FCM	60	0.0675	0.4960	0.5303	0.1188
IFC	60	0.2628	0.2236	0.2893	0.2416
ABC-IFC	60	0.5254	0.7253	0.0510	0.6094

FCM	70	0.0765	0.4920	0.4147	0.1324
IFC	70	0.2671	0.1343	0.2456	0.1787
ABC-IFC	70	0.5984	0.6864	0.0720	0.6394

FCM	80	0.0831	0.4864	0.3930	0.1419
IFC	80	0.3057	0.1777	0.2171	0.2248
ABC-IFC	80	0.5665	0.8331	0.0954	0.6744

FCM	90	0.0894	0.4861	0.4670	0.1510
IFC	90	0.2900	0.2068	0.2246	0.2414
ABC-IFC	90	0.6034	0.7331	0.0561	0.6620

FCM	100	0.0938	0.4829	0.4597	0.1571
IFC	100	0.3136	0.1899	0.2279	0.2366
ABC-IFC	100	0.6081	0.6416	0.0508	0.6244

FCM	110	0.1015	0.3337	0.3986	0.1557
IFC	110	0.3666	0.1956	0.2242	0.2551
ABC-IFC	110	0.6032	0.6785	0.0656	0.6386

FCM	120	0.1073	0.4801	0.3685	0.1754
IFC	120	0.3193	0.1840	0.1903	0.2335
ABC-IFC	120	0.5873	0.6637	0.0479	0.6232

FCM	130	0.1124	0.4819	0.2595	0.1823
IFC	130	0.2813	0.1849	0.1577	0.2231
ABC-IFC	130	0.6118	0.6734	0.0825	0.6411

FCM	140	0.1192	0.4763	0.4354	0.1907
IFC	140	0.2818	0.2373	0.2576	0.2576
ABC-IFC	140	0.5622	0.7579	0.0750	0.6455

FCM	150	0.1303	0.4793	0.4354	0.2049
IFC	150	0.2759	0.1886	0.2456	0.2240
ABC-IFC	150	0.7491	0.7537	0.0830	0.7514

**Table 3:** The proteins classified correctly and wrongly in certain cluster.

Ordinal cluster	The proteins classified correctly				The proteins classified wrongly		
1	YJL154c	YJL053w	YHR012w		—		
2	YLR382c YKR052c	YBR120c YDR194c	YPR134w YIR021w	YOR334w Q0120	YHR005c-a YDL044c	YMR023c YGR222w	YJL133w
3	YDR167w YPL254w YLR055c YHR099w	YDR392w YCL010c YOL148c YDR145w	YGR252w YDR176w YGL066w YBR198c	YGL112c YDR448w YBR081c Y MR236w	—		
4	YNL151c YPR110c YOR116c	YOR224c YHR143w-a YKL144c	YOR210w YOR207c	YPR190c YBR154c	YNL113w	YPR187w	YNR003c
5	YPL218w YAL007c	YIL109c YAR002c-a	YPL085w YAL042w	YML012w	YGL200c YLR208w	YPR181c	YDL195w
6	YOR224c YOR210w YBR154c YPR187w	YDL140c YIL021w YLR418c	YOL005c YDR404c YGL070c YOR151c	YJL140w YHR143w	—		
7	YDR167w YPL254w	YDR392w YCL010c	YGR252w	YGL112c	YDR176w YOL148c YHR099w YMR236w	YDR448w YGL066w YDR145w	YLR055c YBR081c YBR198c
8	YHR069c YDL111c	YDR280w YGR095c	YOL021c YCR035c	YGR195w	—		
